# Effects of respiratory training on pulmonary function, bad mood, and quality of life in patients with COVID-19: A protocol for systematic review and meta-analysis

**DOI:** 10.1097/MD.0000000000026154

**Published:** 2021-06-11

**Authors:** Jianfei Zhu, Qing Long, Huihui Mao, Weirong Ran

**Affiliations:** Department of Nephropathy and Rheumatology, The Central Hospital of Enshi Tujia and Miao Autonomous Prefecture, Enshi, Hubei Province, China.

**Keywords:** Coronavirus Disease 2019, meta-analysis, protocol, respiratory training

## Abstract

**Background:**

At present, whether respiratory training can improve the lung function, quality of life, and mental health of patients with Coronavirus Disease 2019 (COVID-19) is still controversial. Therefore, in order to provide new evidence-based medicine for clinical treatment, we conducted a systematic review and meta-analysis to evaluate the effects of respiratory training in improving lung function, quality of life, and mental health of patients with COVID-19.

**Methods:**

Relevant publications were searched from clinical trials. Computer was used to retrieve Cochrane Central Register of Controlled Trials Repositories, PubMed, Embase, and Web of Science databases. The retrieval time limit was from the establishment of the database to April 2021. Two researchers independently carried out data extraction and literature quality evaluation on the quality and meta-analysis of the included literature was performed with Revman 5.3 software.

**Results:**

The results of this meta-analysis will be submitted to a peer-reviewed journal for publication.

**Conclusion:**

This study will provide reliable evidence-based evidence on the effects of breathing training on lung function, bad mood, and quality of life in patients with COVID-19.

**Ethics and dissemination:**

Ethical approval was not required for this study. The systematic review will be published in a peer-reviewed journal, presented at conferences, and shared on social media platforms.

**OSF Registration number:**

DOI 10.17605/OSF.IO/ZQTGY.

## 1 Introduction

Since December 2019, Coronavirus Disease 2019 (COVID-19) has spread rapidly around the world. COVID-19 patients often suffer from fever, fatigue, and respiratory symptoms such as dyspnea, hypoxemia, and so on.^[1–3]^ Most severe patients developed dyspnea and/or hypoxemia 1 week after the onset of the disease.^[4]^ Severe cases can rapidly develop into acute respiratory distress syndrome, septic shock, metabolic acidosis that is difficult to correct, bleeding and coagulation dysfunction, multiple organ failure, and so on.^[5]^ Respiratory failure is the main cause of death.^[6]^ Therefore, the improvement of respiratory function is very important for COVID-19 patients^[7–9]^ clinically. In addition, from clinic, it was found that there are many bad psychological emotions, including fear, anxiety, and so on.^[10]^ It is characterized by sadness, easy to cry, restlessness, and helplessness, and followed by dizziness, insomnia, loss of appetite, palpitation, chest tightness, and even dyspnea. Therefore, we should also pay more attention to the improvement of patients’ psychological mood, so as to better promote the rehabilitation of COVID-19.

Mainly through a variety of respiratory exercise and treatment techniques, respiratory training can reconstruct the normal respiratory pattern, enhance respiratory muscle function, improve lung ventilation, and reduce dyspnea and improve lung function.^[11–13]^ Respiratory muscle can improve its muscle strength and endurance through exercise, which can prevent respiratory muscle fatigue. At the same time, breathing training is an effective means of rehabilitation exercise. At present, respiratory training is widely applied in the rehabilitation of various respiratory diseases.^[14,15]^

At present, due to insufficient evidence, it cannot be confirmed that respiratory training can improve lung function, bad mood, and quality of life in COVID-19 patients. Therefore, the purpose of this study was to evaluate the effects of respiratory training on pulmonary function, bad mood, and quality of life in COVID-19 patients, thus providing references for non-drug intervention in COVID-19 patients.

## 2 Methods

### 2.1 Protocol

This protocol of systematic review and meta-analysis has been drafted under the guidance of the Preferred Reporting Items for Systematic Reviews and Meta-analysis Protocols.^[16]^ The research framework has been registered on the open science framework (Registration Number: DOI 10.17605/OSF.IO/ZQTGY).

### 2.2 Ethics

Since this is a protocol with no patient recruitment and personal information collection, the approval of the ethics committee is not required.

### 2.3 Eligibility criteria

#### 2.3.1 Types of studies

We will collect all randomized controlled trials (RCTs) and cohort study.

#### 2.3.2 Objects of studies

COVID-19 patients.

#### 2.3.3 Interventions

Patients in the control group were given routine treatment, while patients in the experimental group accepted the treatment of respiratory training on the basis of routine treatment.

#### 2.3.4 Outcome index

Any rating scale describing pulmonary function, mood, and quality of life.

### 2.4 Exclusion criteria

(1) Studies without a control group.
(2) Review articles, techniques, case reports, letters to the editor, and editorials are excluded.

### 2.5 Search strategy

The literature retrieval was carried out online from Clinical Trials. Computer was used to retrieve Cochrane Central Register of Controlled Trials Repositories, PubMed, Embase, and Web of Science databases. The retrieval time limit was from the establishment of the database to April 2021. Taking PubMed as an example, the retrieval strategy is displayed in Table 1.

**Table 1:** PubMed search strategy.

Number	Search terms
#1	Respiratory training[Title/Abstract]
#2	Breath training[Title/Abstract]
#3	Breathing exercise[Title/Abstract]
#4	Respiratory rehabilitation training[Title/Abstract]
#5	or/1–4
#6	Corona Virus [Title/Abstract]
#7	Corona Virus Disease 2019 [Title/Abstract]
#8	COVID-19 [Title/Abstract]
#9	Novel coronavirus[Title/Abstract]
#10	Novel coronavirus pneumonia[Title/Abstract]
#11	or/6–10
#12	#5 AND #11

### 2.6 Data screening and extraction

Two authors independently extracted the data. Disagreements were resolved through discussion until a consensus was reached or consultation with a third author.

The contents extracted from each paper are as follows:

(1) study information: the authors and the year of publication;
(2) the number of cases in the experimental group and control group;
(3) study indicators, including various lung function, mood, and quality of life scale scores;
(4) baseline characteristics including sex, age, and severity of the disease, etc.

**Figure 1:**
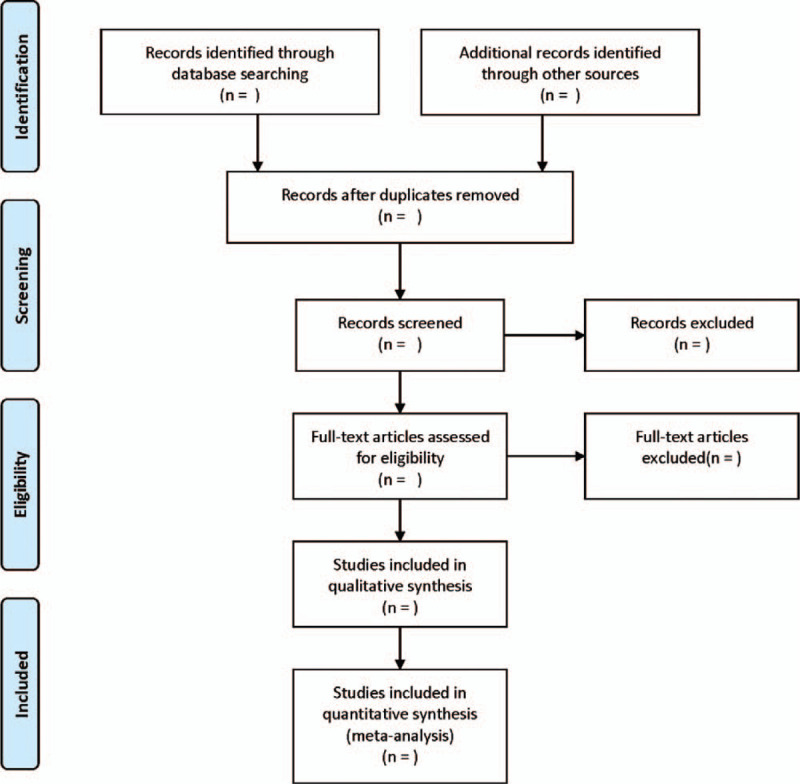
Flow diagram of literature retrieval.

The literature selection process is listed in Figure 1.

### 2.7 Quality evaluation

The risk bias assessment tool will be built into Review Manager 5.3 software (the Cochrane collaboration's tool for assessing risk of bias) and will be adopted to evaluate the risk bias of the included RCTs. The quality scores of the non-RCTs were evaluated by the Newcastle-Ottawa Scale.^[17]^

### 2.8 Statistical analysis

Rev-Man 5.3 statistical software was used for the meta-analysis. The chi-square test was performed to test the heterogeneity of the included studies. If *P* ≥ .1 and *I*^2^ ≤ 50%, there was no statistical heterogeneity among the results of the studies, and a fixed-effect model (Mantel-Haenszel method) was adopted for analysis, or a random-effect model was used. Continuous variables are extracted and analyzed to mean value ± SD. Standardized mean differences with a 95% confidence interval are assessed for continuous outcomes. *P* < .05 was statistically significant.

#### 2.8.1 Dealing with missing data

If there are missing data in the article, the corresponding author or the first author will be contacted through email to obtain accurate data. If the author cannot be contacted, or the author has lost relevant data, descriptive analysis will be conducted instead of meta-analysis.

#### 2.8.2 Subgroup analysis

According to the duration of intervention and severity of illness, subgroup analysis will be carried out.

#### 2.8.3 Sensitivity analysis

In order to judge the stability of the outcome index, sensitivity analysis will be conducted to analyze each outcome index.

#### 2.8.4 Publication bias

If the number of studies included in an outcome indicator is no less than 10, a funnel chart will be used to assess publication bias.^[18]^ In addition, Egger's and Begger's tests will be carried out to evaluate potential publication bias.

## 3 Discussion

Characterized by fast transmission, strong infectivity and general susceptibility of the population, COVID-19 is a fulminant epidemic infectious disease that endangers public health.^[19–21]^ The main clinical manifestations include fever, cough, and fatigue, and some patients are accompanied by headache, dizziness, muscle soreness, diarrhea, and other discomfort, and gradually develop dyspnea, chest tightness, and suffocation.^[22]^ There is evidence that breathing training can effectively promote lung rehabilitation, relieve bad mood, and improve the quality of life.^[23–25]^ On the contrary, to the best of our knowledge, there is no meta-analysis to investigate respiratory training interventions in COVID19 patients to improve their mental health and lung function. Therefore, in order to provide new evidence of evidence-based medicine for clinical treatment, we conducted a systematic review and meta-analysis to evaluate the effects of respiratory training on lung function, bad mood, and quality of life in COVID-19 patients. This paper is a protocol of systematic evaluation and meta-analysis, and the evaluation tools are described in details. For this study, our audit process will be very strict. The results of our review will be reported in strict accordance with PRISMA standards. Moreover, the results of this meta-analysis will provide evidence-based basis for clinical lung rehabilitation guidance.

## Author contributions

**Conceptualization:** Weirong Ran, Jianfei Zhu.

**Data collection:** Qing Long.

**Data curation:** Weirong Ran, Jianfei Zhu, Qing Long.

**Formal analysis:** Qing Long.

**Funding acquisition:** Weirong Ran.

**Funding support:** Weirong Ran.

**Investigation:** Qing Long.

**Methodology:** Qing Long.

**Project administration:** Weirong Ran.

**Resources:** Huihui Mao.

**Software operating:** Jianfei Zhu.

**Software:** Huihui Mao.

**Supervision:** Weirong Ran.

**Validation:** Huihui Mao.

**Visualization:** Huihui Mao.

**Writing – original draft:** Jianfei Zhu and Weirong Ran.

**Writing – review & editing:** Jianfei Zhu and Weirong Ran.
